# Healthy aging delays the neural processing of face features relevant for behavior by 40 ms

**DOI:** 10.1002/hbm.24869

**Published:** 2019-11-29

**Authors:** Katarzyna Jaworska, Fei Yi, Robin A. A. Ince, Nicola J. van Rijsbergen, Philippe G. Schyns, Guillaume A. Rousselet

**Affiliations:** ^1^ Institute of Neuroscience and Psychology University of Glasgow Glasgow UK

**Keywords:** aging, EEG, face processing, information processing, mutual information

## Abstract

Fast and accurate face processing is critical for everyday social interactions, but it declines and becomes delayed with age, as measured by both neural and behavioral responses. Here, we addressed the critical challenge of understanding how aging changes neural information processing mechanisms to delay behavior. Young (20–36 years) and older (60–86 years) adults performed the basic social interaction task of detecting a face versus noise while we recorded their electroencephalogram (EEG). In each participant, using a new information theoretic framework we reconstructed the features supporting face detection behavior, and also where, when and how EEG activity represents them. We found that occipital‐temporal pathway activity dynamically represents the eyes of the face images for behavior ~170 ms poststimulus, with a 40 ms delay in older adults that underlies their 200 ms behavioral deficit of slower reaction times. Our results therefore demonstrate how aging can change neural information processing mechanisms that underlie behavioral slow down.

## 1 INTRODUCTION

**Figure 1:**
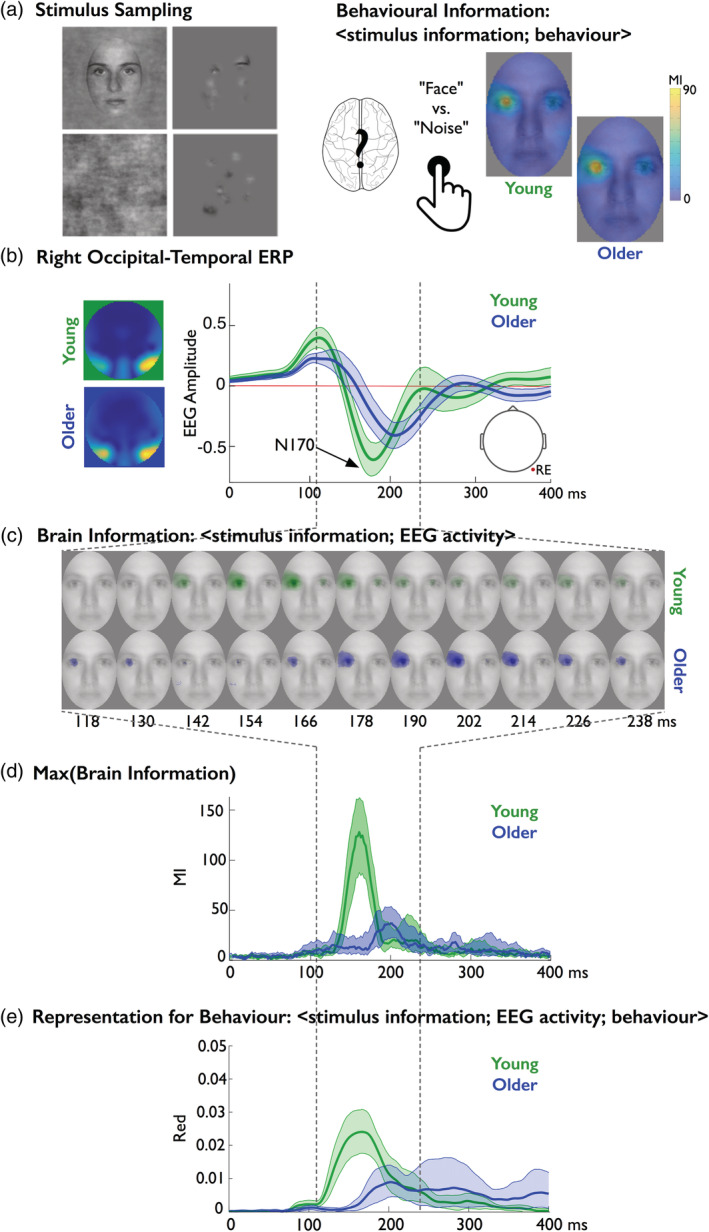
Illustration of the experimental paradigm and analyses. (a) Stimuli. Eighteen young and 19 older participants each detected faces versus noise textures from 2,220 pictures that revealed information through Gaussian apertures (“Bubbles” Gosselin & Schyns, 2001), while we recorded their face detection behavior (with key presses) and electroencephalogram (EEG) brain activity. We performed the following computations in each individual observer. (b) Behavioral information. We used mutual information (MI) to compute <stimulus information; behavior>, the relationship between stimulus pixel visibility on each trial and the corresponding reaction times (RTs). The same left eye of faces was associated with faster RTs in both young and older participants. (c) Event‐related potentials (ERPs). Based on these trials, a full brain analysis revealed larger average ERPs associated with face detection at the right hemisphere occipital‐temporal electrode (right electrode [RE], whose location is sketched with a cranial view of the head underneath) of both young and older adults. (d) Brain information. Using MI, we computed <stimulus information; EEG activity> every 2 ms poststimulus, which represents the relationship between stimulus pixel visibility in a single trial and the corresponding electrode voltage amplitudes, to reveal the dynamics of any visual feature represented in the variations of the EEG. For illustration, we plotted examples of these classification images every 12 ms between 118 and 238 ms poststimulus. The resulting MI images revealed that the N170 of both young and older adults represented the same left eye, although older adults did so with a delay. (e) Max (brain information). To precisely estimate this age‐related, feature‐processing delay, we plotted the time course of maximum MI (across the pixels of each MI image in (d)) between 0 and 400 ms poststimulus, which peaked 40 ms later in older participants. (f) Representation for behavior. Finally, we confirmed that these features are represented in the brain to support face detection behavior. To do this, we computed feature redundancy (*FeatRed*), which quantifies the common effect of stimulus information on both EEG activity and behavior from the triple relationship <stimulus information; EEG activity; behavior>

There is strong evidence that an age‐related slowing down occurs when performing various behavioral tasks (Salthouse, 2000). There is also parallel evidence that age‐related changes in the brain can slow down neural processing (Gazzaley et al., 2008; Nakamura et al., 2001; Rousselet et al., 2010, 2009; Wiese, Schweinberger, & Hansen, 2008). Although these studies can inform where and when, in the brain, aging can impact neural activity, a critical challenge remains to develop theories of cognitive aging that explain how the neural information processes that are involved in a cognitive task contribute to slow down behavior. It is imperative to address this challenge to understand how aging impacts the specific cognitive mechanisms that mediate behavior. To illustrate, consider the fundamental social cognition task of detecting a face (see Figure 1). Although we expect older participants to detect faces in this task more slowly, a more complete understanding of the effects of aging on cognitive processing requires richer data. At a minimum, we need to characterize the face information that young and older participants selectively use when detecting faces, for example, because slower face detection could result from older adults needing more facial features, or simply because older participants do not use the same features. We also need to characterize the information content of the neural activity underlying the behavioral task at hand, because the age‐related slowing could arise from neural representation of several task‐irrelevant features (a decline of selectivity). Finally, we would also need to trace the origin of the behavioral delay in the neural mechanisms that represent the face for the detection task per se, for example, because older brains could be generally delayed in their onset of neural activity when responding to any visual stimuli, rather than specifically delayed when processing the task‐relevant face features.

In this study, we present a new paradigm to advance and deepen our understanding of information processing in cognitive aging. We developed stimulus information representation (SIR), an information theoretic framework, to tease apart stimulus information that supports behavior from that which does not. This new framework considers the interactions between three important variables, not just two, as is the norm: stimulus information, neural electroencephalogram (EEG) activity, and behavior. We used the Bubbles technique (Gosselin & Schyns, 2001) to randomly sample visual information from the stimulus on each trial (see Figure 1a), which limits researcher bias by not making a priori assumptions about the features that participants in different groups use for the task. With the double relationship <stimulus information; behavior>, we coupled the stimulus information sampled on each trial with the corresponding participant detection behavior to reconstruct the features that underlie their detection behavior (see Figure 1b). With the double relationship <stimulus information; EEG activity>, we coupled the same sampled information with the corresponding EEG activity of each participant's brain engaging with the detection task, to independently characterize where, when and how brain activity represents face features (see Figure 1c–e). Finally, with the new triple relationship <stimulus information; EEG activity; behavior>, we directly visualized with a single integrated measure the dynamic development of the representation of a face in the brain to support face detection behavior (see Figure 1f). We computed the dynamics of representation individually in 17 young and 18 older participants and showed, using information theoretic redundancy across participants, that delayed behavioral information processing and delayed reaction time (RT) reflect a common aging factor within our sample. We now present each double relationship in turn, followed by the triple relationship, and the group redundancy analysis. Section titles indicate the specific relationship that we evaluate between the three variables.

## 2 RESULTS

### 2.1 Behavioral information: <stimulus information; behavior>

**Figure 2:**
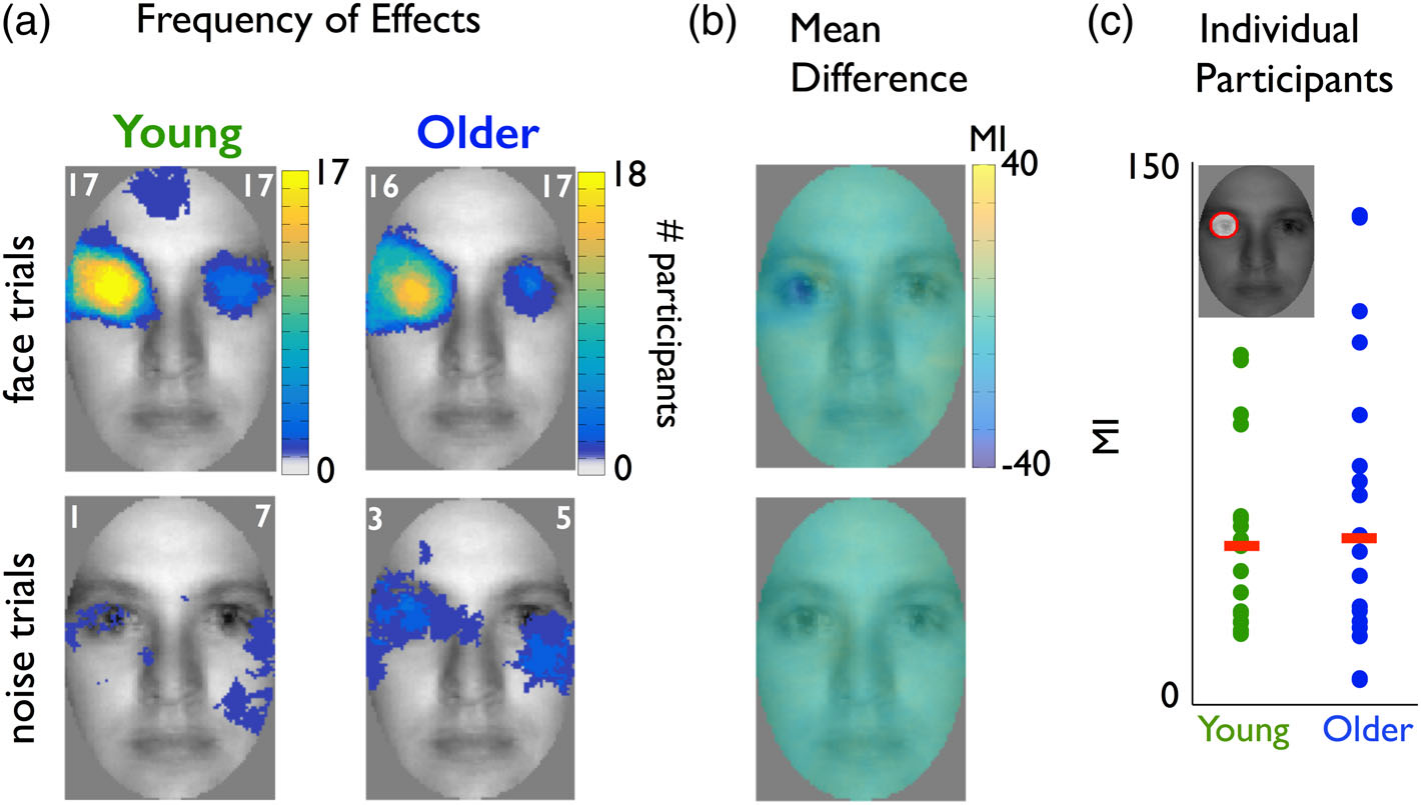
Behavioral information. (a) Significant effects. The white number in the left (vs. right) upper corner of each face image indicates the maximum number of participants showing a significant relationship between pixel visibility and RT at the same (vs. any) face pixel. Yellow colors reflect that a high number of significant effects across participants cluster in the left eye region of the image in young and older participants. As such, for most young and older participants, the left eye of the face image was associated with faster RTs (see also Supplementary Results). (b) Mean difference. Images show the differences between average mutual information (MI) images (young–older) for face trials (top) and noise trials (bottom). The scale represents normalized MI values (see Section 4). (c) Individual participants. Dot plot shows, for each participant (colored dot), MI values summed within a mask that captures left eye pixels (represented as a red circle in the face inset; see *Supplementary Methods*). Red bars correspond to the median of these per‐participant averages. Distributions of individual participant values were similar between the two groups

Seventeen young (median age = 23) and 18 older (median age = 66) participants categorized 2,200 pictures of faces and noise textures revealed through Gaussian apertures (so‐called “Bubbles,” Gosselin & Schyns, 2001), which sample random spatial regions of face and noise images on each trial. Young participants were 198 ms faster to detect faces (median RT in young = 378 ms, 95% confidence interval = [349, 401] vs. RT in older participants = 576 ms [527, 604]). To determine the relationship between sampled face information (pixels) and the varying RTs on each trial, we computed mutuaI information (MI) between <stimulus information; RT > (see Section 4). We found that the presence of the left eye in image samples modulated RTs in all young participants (17/17) and in most older participants (16/18; see Figure 2). The presence of the right eye also modulated RTs in a few young and older participants. However, whereas young participants could use many other features to perform the task accurately, older participants specifically used the eyes (see Supplementary Results and Supplementary Figures S1–S4).

### 2.2 EEG face information representation: <stimulus information; EEG activity>

**Figure 3:**
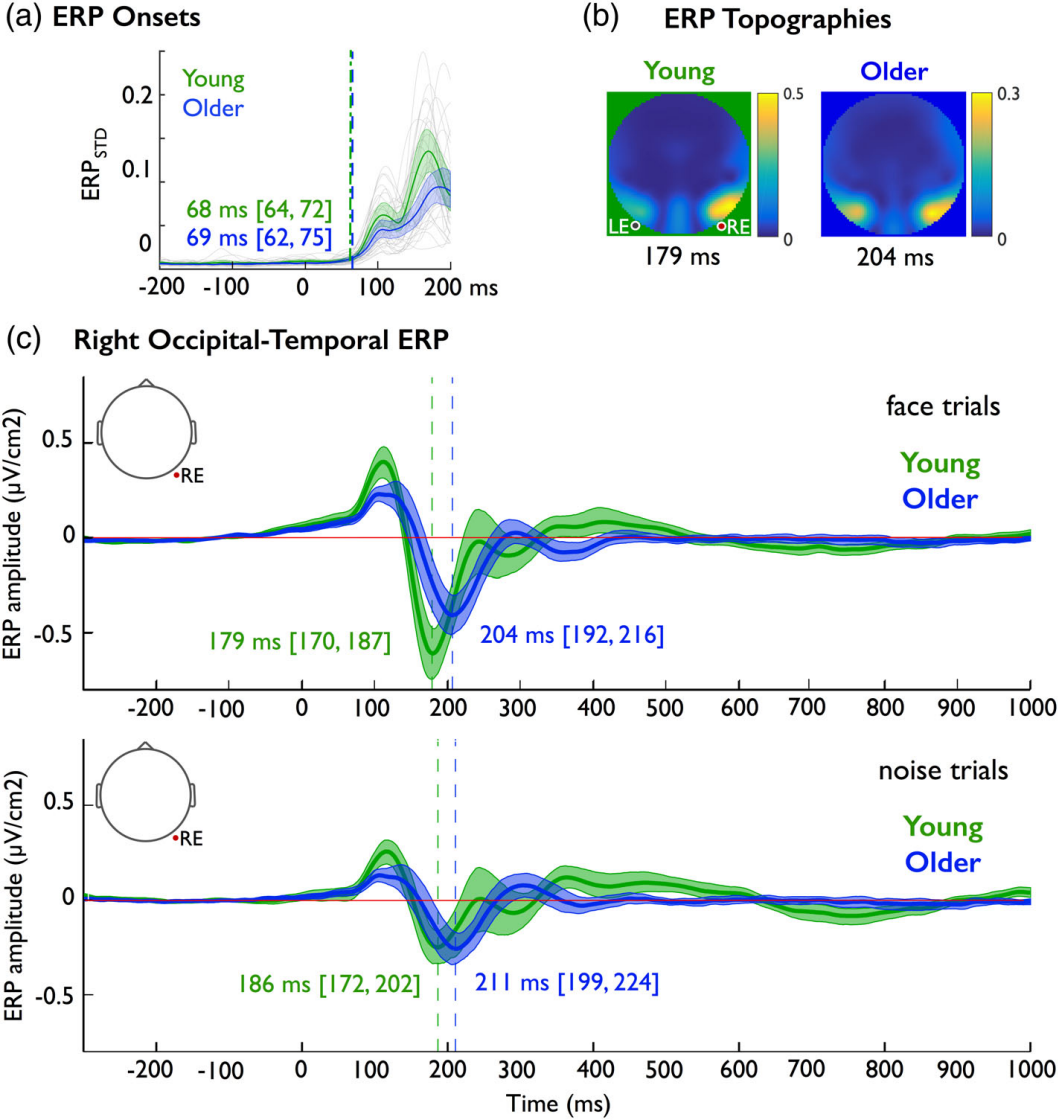
N170 event‐related potentials (ERPs). (a) ERP onsets. Thin gray lines show individual participants' ERP_STD_ (μV/cm^2^). Colored thick lines show group averages with shaded areas indicating 95% confidence intervals around the group means. Vertical dashed lines mark the overlapped onset times of cortical activity in each group. (b) ERP topographies. For face only trials, we averaged across participants the largest squared ERP amplitude at the time of its peak. (c). Right occipital‐temporal ERP. Thick lines show averaged ERPs across young (green) and older (blue) participants, for face (top panel) and noise bubble trials (bottom panel), with shaded areas corresponding to 95% confidence intervals. In each panel, numbers indicate the group median (and confidence intervals) of N170 latencies, which are delayed by ~20 ms in older adults

We now turn to the important question of where, when and how brain processes represent task‐relevant features to support behavioral decisions. Before we proceed, we must first rule out low‐level optical factors as the main contributor to any age‐related delay in our analyses. To do this, we computed the time course of the standard deviation of the mean ERP across electrodes (ERP_STD_) using causal‐filtered data (see Section 4 and Figure 3a). ERP_STD_ onsets correspond to the initial activation of the occipital cortex that follows stimulus presentation (Foxe & Simpson, 2002), which could reveal generic, age‐related delays already present in the earliest stages of the visual processing pathway. However, we found similar ERP_STD_ onsets in young (68 ms [64, 72]) and older (69 ms [62, 75]) participants (Figure 3a), with a negligible difference between them (−0.5 ms [−7, 5]), which suggests that there is no clear evidence for a generic delay in the onset of cortical activity in response to visual stimuli in older participants.

#### 2.2.1 N170 is delayed in older participants

Despite no clear generic neural delay in older participants, their peak N170 response latencies to full faces on the right occipital electrode (RE) were delayed relative to the N170 response of young participants by 18 ms [9, 24] (and by 23 ms [9, 38], in response to the practice full noise trials; see Section 4.2). These N170 peak delays on the RE were confirmed in bubbles trials that sampled face and noise information (i.e., 22 ms [10, 32] and 18 ms [7, 31], respectively, see Figure 3c; see Figure 3b for a summary topography). The N170 latency delay on bubble noise trials was unlikely to be attributable to false alarms. Analysis of behavioral responses revealed that older adults were less accurate than young adults only on face trials (i.e., had more “misses”; see also Supplementary Results), whereas both groups were highly accurate on noise trials.

N170 peak amplitudes were similar in young and older participants, except on practice noise trials, where they were larger in older than young participants (see Supplementary Results and Supplementary Figure S5).

#### 2.2.2 Contralateral eye representation over the N170 time course

Our novel methodology enables us to directly visualize the dynamic representation of facial features in the single‐trial amplitude variations of EEG activity during the detection task. To do this, we computed the MI relationship between <pixel visibility information; EEG activity>, for each stimulus pixel, on the left (LE) and right (RE) electrode (see Section 4.5), every 2 ms between 0 and 400 ms post‐stimulus. Figure 1d illustrates example MI classification images of brain information on electrode RE.

**Figure 4:**
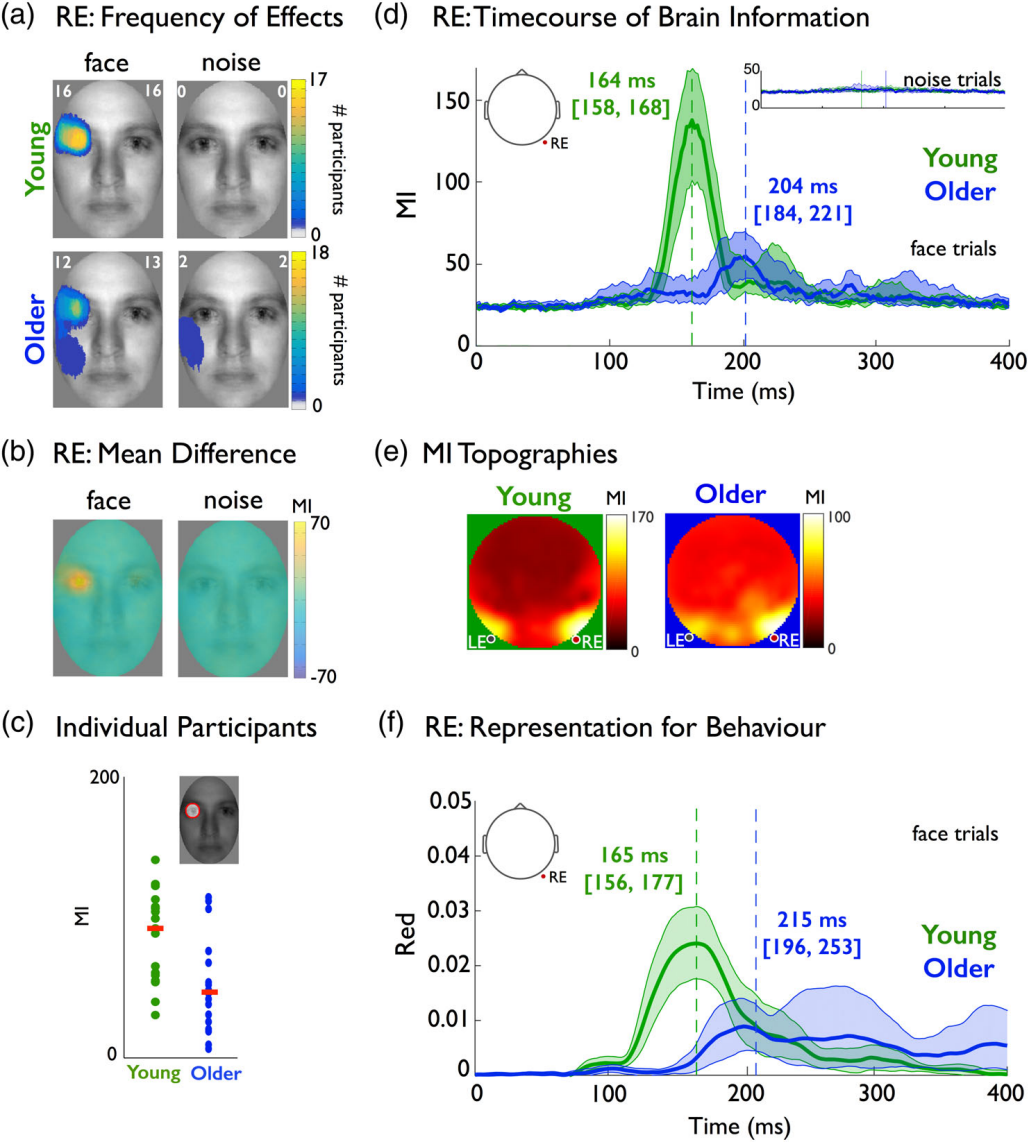
Brain information. (a) Right electrode (RE): frequency of significant effects. Using the maximum mutual information (MI) image across time points on RE in each participant, the white number in the left upper corner of every image corresponds to the maximum number of participants showing a significant effect at the same face pixel (on face and noise trials separately), whereas the number in the right upper corner corresponds to the total number of participants showing significant effects at any pixel. (b) RE: mean difference. Averaging the maximum MI images across young and older participants separately produced images of group differences (young–older) for face trials (left) and noise trials (right). Average MI in the left eye region of the face image was higher for young than older participants. Throughout the figure, MI represents normalized MI values (see Section 4). (c) Individual participants. Dot plot shows, for each participant (colored dot), MI values summed within a mask that captures left eye‐only pixels (represented as a red circle in the face inset; see *Supplementary Methods*). Red bars correspond to the median of these per‐participant averages. Distributions of individual participant values were different between the two groups. (d) RE: time course of brain information are presented for both face and noise (insets) bubbles trials on RE. Color‐coded numbers correspond to median latencies of maximum MI in both groups. Shaded areas correspond to bootstrap 95% confidence intervals around the 20% trimmed mean. (e) MI topographies. MI <face information; electroencephalogram (EEG) activity> (i.e., brain information) averaged across young and older participants for each electrode across time points and face pixels. (f) Right electrode (RE): representation for behavior. A time course of behavioral redundancy shows a 46 ms delay in the representation of the left eye on the RE for delayed face detection reaction time (RT) in older adults

As shown in Figure 4a, EEG activity at RE contralaterally represented the left eye in most young (*N* = 16/17) and older (*N* = 12/18) adults (see Supplementary Figures S7 and S8 for LE results), with a weaker overall representation in older adults (their peak MI was 57% [42, 82] of that of young adults, see Figure 4b,c).

We then used the resulting images of brain information to compute their maximum MI across all time points and pixel locations (see Figure 4d). Peak EEG representation of the left eye occurred 40 ms [23, 57] earlier in young adults relative to older adults (i.e., ~164 ms in young vs. ~204 ms in older adults).

To rule out the possibility that maximum information was represented on other electrodes, we independently performed a full brain‐by‐time analysis. To this end, we again computed the MI relationship between <pixel visibility information; EEG activity> at each pixel and time point between 0 and 400 ms poststimulus, on all electrodes. We found that maximum MI peaked primarily in the left and right lateral‐occipital region in both young and older observers (see Figure 4e). Furthermore, computing the maximum MI across all electrodes revealed similar results to those reported here for RE (see Supplementary Figure S6).

At this juncture, it is important to emphasize that we also ruled out a potential effect of participants paying spatial attention to the location of the eyes in sample images (rather than one of representation of the eyes per se) by repeating the above analyses using only noise trials. In these noise trials, we did not find sensitivity to the image locations of the eyes (i.e., where the eyes of a face would be in face trials), in any participant. Note that the midline electrode (Oz, see Supplementary Figures S7 and S8) also revealed the weaker representation of various other facial features (such as eyes, chin, mouth, nose, and forehead) in some participants. As these representations were inconsistent across participants, we did not analyze them further.

### 2.3 Stimulus representations in the brain to support behavioral decision: <stimulus information; EEG activity; behavior>

So far, we have shown that face detection behavior in all participants involves the processing of eyes in the stimulus (particularly the left one), with a ~200 ms decision response delay in the older group. We have also shown that this left eye impacted neural EEG responses in most participants, with a 40 ms representation delay in the older group. Now, we integrate behavioral and brain results by showing where the representation of the eyes in the EEG also modulates face detection behavior, on the same trials.

To do this, we computed separately for each participant the information theoretic feature redundancy (Ince et al., 2017), red (eye; EEG; RT), which is the shared variability between three variable: single‐trial visibility of the left and right eye in the stimulus, at contralateral electrodes (RE and LE), together with the corresponding RTs (see Section 4.10). In this application, redundancy measures the strength of the representational similarity of a given stimulus feature (e.g., relative visibility of the left eye) between variations of EEG amplitude (e.g., on the RE) and variations of face detection RT. On the RE, we found a 46 ms [30, 81] redundancy delay between the young and older participants (i.e., 165 ms peak [155, 178] for young, and a 215 ms peak [196, 246] for older adults; see Figure 4f). We observed a similar 42 ms [20, 86] delay between young and older participants on the LE, where right eye redundancy peaked at 172 ms [161, 186] for young, and at 220 ms [196, 261] for older adults (see Supplementary Figure S10). These results confirm that the EEG and RT similarly represent the eyes, but with a 42 ms delay in older adults.

### 2.4 Delayed stimulus representations in the brain underlie delayed behavioral RTs

We now demonstrate that the delayed representation of the eyes in the EEG underpins delayed RT in older adults. We tested this by computing the redundancy between the age groups, RT latency, and the peak latency of EEG eye representation (see Section 4.11). Specifically, we first quantified (with MI) the strength of the relationship between age group and each participant's median RT—it was 0.5 [0.36 0.81] bits—and also between age group and redundant eye representation latency in the EEG—it was 0.28 [0.08 0.64] bits for the left eye (at RE) and 0.29 [0.08 0.69] bits for the right eye (at LE). Having shown that both RT latency and EEG eye representation associate with age group, we asked whether these two associations themselves overlap—i.e., whether we can similarly predict age group from RT latency and EEG eye representation. We found that the redundancy between latency of the left eye representation (at RE) and RT was 0.28 [0.06 0.51] bits. This was 100% of the age prediction obtained from the left eye representation alone (0.28 [0.08 0.64]). We also found that redundancy between latency of the right eye (at LE) and RT was 0.12 [−0.09 0.43] bits. This was 40% of the age prediction obtained from the right eye representation alone (0.29 [0.08 0.69]). These results demonstrate that only the left eye (at RE) representation is purely related to the RT deficit between age groups. The results also reflect the strongly lateralized relationship between stimulus and RT (Figure 2).

To summarize, we used information theoretic redundancy at two levels (within and across participants) to demonstrate a direct link between behaviorally relevant neural information processing change (48 ms delay in left eye redundancy) and a behavioral aging deficit (200 ms RT delay across participants of our sample). Specifically, within participants, we explicitly quantified the triple relationship <stimulus information; EEG activity; behavior> within SIR to focus on the aspects of information processing reflected in EEG signals that are directly relevant to face detection RT. Across participants, quantification of the triple relationship <age group; representational delay; RT delay> suggests a group level interpretation in which right‐lateralized (but not left‐lateralized) neural representational delays underlie behavioral slowdown in aging.

## 3 DISCUSSION

Here, we set out to investigate how information‐processing delays in the aging brain could slow down face detection behavior (a fundamental social interaction task), using the novel information theoretic measures of the SIR framework. Specifically, we considered the interactions between three variables: stimulus information, neural EEG activity, and RT, and compared the results between young and older adults. We characterized the face information that young and older participants selectively use when detecting faces (i.e., the eyes of a face), and we traced the origin of the behavioral delay with aging to a delay in the neural processes that represent the eyes of faces for the task during the N170 period. We ensured that the neural eye representation delay could not be explained by generic delays of onsets of cortical activity in young and older participants.

This study provides an important step toward understanding visual cognitive aging. First, we demonstrate that young and older adults can use the same face information (the eyes) to quickly detect faces. As such, our results contrast with previously published findings that suggest that a differential use of horizontal versus vertical information might underlie the impairment of older adults when identifying faces (Chaby, Narme, & George, 2011; Obermeyer, Kolling, Schaich, & Knopf, 2012). Here, although older adults had fewer correct responses when detecting faces, the same face information modulated their slower RTs. Our analyses of correct versus incorrect responses (see Supplementary Results) revealed that while young adults can use any facial features to detect a face, older adults relied heavily on the eyes for correct detection. As such, it is possible that rather than inefficiently extracting information from faces, older adults were more conservative, detecting a face only when its eyes were visible (see also van Rijsbergen, Jaworska, Rousselet, & Schyns, 2014), although another possibility is that older adults relied more on local contrast information contained within the eye region of the face, in line with previous studies showing that they require more contrast to detect and discriminate faces (Lott, Haegerstrom‐Portnoy, Schneck, & Brabyn, 2005; Owsley, Sekuler, & Boldt, 1981). In any case, the uncovering of such difference was made possible by Bubbles sampling (Gosselin & Schyns, 2001), which limits researcher bias by not making a priori assumptions about the features that participants should use (Creighton, Bennett, & Sekuler, 2018; Éthier‐Majcher, Joubert, & Gosselin, 2013; van Rijsbergen et al., 2014).

Establishing equivalence of behavioral information in the two groups is an important benchmark for comparing the task‐relevant neural coding of information. Here, we found that the EEG activity of both young and older adults represented the eye pixels contralateral to the lateral‐occipital recording electrodes, an effect that was stronger at the right hemisphere electrodes in both groups, in agreement with the reported right hemisphere dominance for face processing (Sergent, Ohta, & MacDonald, 1992). Although feature representation was qualitatively similar across young and older adults, it was delayed by 40 ms and weaker in older adults, in the absence of generic delays in the onset of visual cortical activity in older participants. This suggests that the reported delay occurred at the stages of cortical information processing, and was not due to precortical neural factors; thereby adding to the evidence that processing speed delays are unlikely to be due to bottom‐up optical factors, such as senile miosis, contrast sensitivity (Bieniek, Bennett, Sekuler, & Rousselet, 2016; Bieniek, Frei, & Rousselet, 2013), or visual acuity (Price et al., 2017). Furthermore, we believe that bottom‐up optical factors were unlikely contributors to the observed differences at the neural level, because any bottom‐up factors should affect all neural responses irrespectively of their category, whereas we observed much larger N170 to noise textures in older than in young participants.

Although our stimuli were only frontal views of faces, it would be interesting to test side views, in line with previous studies showing age‐related behavioral decrement on perception of faces across viewpoints (Habak, Wilkinson, & Wilson, 2008; Wilson, Mei, Habak, & Wilkinson, 2011). Processing of facial/head viewpoints follows a distinct sequence of encoding that reflects different levels of computational complexity (Kietzmann, Gert, Tong, & König, 2017). Whether aging affects different stages of viewpoint processing similarly would inform whether processing speed is delayed in a constant or cumulative manner along the visual cortical hierarchy (Price et al., 2017).

The age‐related delay that we report here agrees with previous cross‐sectional results, which suggest that face processing slows down from 20 years of age and onwards (Bieniek et al., 2013; Rousselet et al., 2010, 2009). However, previous studies could not ascribe these delays to the representation of task‐relevant features, as reported here (see also Rousselet, Ince, van Rijsbergen, & Schyns, 2014; Schyns, Petro, & Smith, 2007; Smith, Gosselin, & Schyns, 2004). Such task‐relevant feature representations in the brain are important for two main reasons. First, by demonstrating the neural representation of task‐relevant face features in older adults, we can show that the reported delays (neural and behavioral) do not arise from an inability to inhibit the task‐irrelevant information that increases with age (Gazzaley et al., 2008; Zanto, Toy, & Gazzaley, 2010). Second, we can use the direct evidence of neural representation of the same features to demonstrate that the N170 time window is functionally equivalent in young and older adults (cf. Rousselet et al., 2010). The N170 is an early component of face processing (Bentin, Allison, Puce, Perez, & McCarthy, 1996; Itier, Alain, Sedore, & McIntosh, 2007; Rousselet et al., 2014) that is delayed in older participants (Gazzaley et al., 2008; Nakamura et al., 2001; Rousselet et al., 2009; Wiese et al., 2008). Comparing the latencies from the same component across age groups presumes that it indexes the same neuronal processes over a life span. However, prior to this current study, it was unclear whether the single‐trial activity evoked during the N170 time window represents the same information content across age groups. Here, we show that the N170 performs the same representation function in two age groups detecting faces, albeit with a delay in older adults (Rousselet et al., 2009; Schyns et al., 2007; Smith et al., 2004; van Rijsbergen & Schyns, 2009). In fact, our results suggest that the 40 ms representation delay of the eyes over the N170 in older adults was directly related to their 200 ms RT delay when using this information to detect faces. We can thus trace the origin of the behavioral slowing to the early stages of stimulus processing, which could complement other studies that have indicated that slowing originates from motor response generation (Kolev, Falkenstein, & Yordanova, 2006; Yordanova, Kolev, Hohnsbein, & Falkenstein, 2004).

At this juncture, it is important to emphasize that the age‐related delay in the processing of the eye information from the sample image cannot be attributed to the presence of Bubble masks. Bubbles can be thought of as a “masking procedure” that degrades the visual input and possibly entails object completion (Tang et al., 2014). The processing of occluded stimuli by the visual system might require additional resources to perform the task, leading to longer processing times (Sekuler, Gold, Murray, & Bennett, 2000). As such, any delay observed in a sample of older adults could be due to a combination of factors: a genuine slowing down of processing speed, as well as an increase in the time needed to process the occluded stimulus with respect to young adults. However, our ERP results show that the extra processing time of Bubbled images compared with full images differed very little between young and older participants. Specifically, even though the processing of the Bubbled stimuli was delayed with respect to full images by about 20 ms in both young and older participants, there was only a weak interaction between age and masking condition. In both practice (unmasked) and Bubble (masked) trials, the N170 latency to face images in older participants was delayed by about 20 ms (18 ms in practice trials and 22 ms in Bubble trials) with respect to that in young participants. This agrees with a recent study (Bieniek et al., 2013), which showed that even though stimulus luminance affects the entire ERP time course in both young and older participants, it does not affect age‐related differences in processing speed.

Whether other stimulus characteristics (e.g., external features, color pigmentation) affect processing speed and use of diagnostic information with age remains to be explored. Here, we used relatively sparse images of grayscale faces, presented in an oval mask, without any external features such as hair. Additional information such as color might increase the diagnostic information across the whole face, whereas external features might become more relevant at longer viewing distances. Furthermore, we only used images of young faces. Aging is associated with changes to the physical appearance of the face (e.g., wrinkling, pigmentation; van Rijsbergen et al., 2014) that might affect the diagnostic information for a particular task. While face processing strategies might vary depending on the age of the observer relative to the age of the stimuli shown (own‐age bias; van Rijsbergen et al., 2014; Anastasi & Rhodes, 2005; Wiese et al., 2008), we do not know whether these will affect N170 latencies or amplitudes (Komes, Schweinberger, & Wiese, 2015; Wiese et al., 2008).

Altogether, future studies should aim to uncover the extent to which delays in other processing stages (i.e., postperceptual decision, sensorimotor integration, or motor generation processes) influence the observed behavioral slow down. Our results also raise further considerations regarding their generalization to other stimulus categories, both simple and complex, such as scenes, objects, words, but also sensory modalities and tasks. Recent evidence suggests that aging cannot be regarded as a unitary concept that affects functionally relevant brain regions in the same manner (Price et al., 2017). Instead, whether the observed age‐related delay is constant or cumulative, may depend on a variety of factors other than structural differences in brain regions (Peters, 2009; Price et al., 2017; Raz et al., 2005; Wang, Zhou, Ma, & Leventhal, 2005), such as the sensory modality, task, and stimulus complexity. Our new methods can address some of these issues by linking tightly controlled stimulus information to brain response and decision behavior in important social cognition tasks. These methods can also be extended to other sensory modalities to study group differences (i.e., cultural, developmental, clinical) in perception and cognition.

To summarize, our results provide the first functional account that advancing age involves differences in neural delays in the representation of task‐relevant information for behavior. They address the fundamental challenge of developing theories of cognitive aging that explain how the neural information processes involved in a cognitive task slow down behavior.

## 4 METHODS

### 4.1 Participants

Eighteen young (nine females, median age = 23, min 20, max 36) and 19 older adults (seven females, median age = 66, min 60, max 86) participated in the study (data of 15 of the young participants were used in Rousselet et al. (2014)). Only one participant was >80 years, the age range of all other older adults was 60–77, comparable to that of the young adults. In a face discrimination task, we have shown a qualitative shift in the time course of brain activity around 47 years of age (Rousselet et al., 2010), which we investigated here with age ranges appropriate for the research question.

All older adults were local residents. We excluded participants if they reported any current eye condition (i.e., lazy eye, glaucoma, macular degeneration, cataract), had a history of mental illness, were currently taking psychotropic medications or used to take them, suffered from any neurological condition, had diabetes, or had suffered a stroke or a serious head injury. We also excluded participants if their latest eye test was more than a year (for older participants) or 2 years (for young participants) prior to the study taking place. Two older participants reported having cataracts removed, and one older participant reported having undergone a laser surgery. We included them because their corrected vision was within normal range. In addition, older participants completed the Montreal Cognitive Assessment to screen for cognitive impairment. All participants achieved a score of 26 or above, indicating normal cognitive performance. We assessed all participants' visual acuity and contrast sensitivity in the lab using a Colenbrander mixed contrast card set and a Pelli–Robson chart. All participants had normal or near‐normal visual acuity as measured with the 63 cm viewing distance (computer distance) chart (Table 1). Three older participants had contrast sensitivity of 1.65, and all others had contrast sensitivity of 1.95 log units, within the normal range of contrast sensitivity for that age group (Elliott, Sanderson, & Conkey, 1990). All young participants had contrast sensitivity of 1.95 log units or above. During the experimental session, participants wore their habitual correction if needed.

**Table 1:** Visual test scores. Visual acuity scores are reported for HC and LC charts presented at the 63 cm viewing distance, and expressed as raw VAS

	HC 63	LC 63	CS
Young	108 [95, 110]	99 [94, 104]	1.95 [1.95, 2.25]
	−*0*.*16* [*0*.*10*, −*0*.*20*]	*0*.*02* [*0*.*12*, −*0*.*08*]	
Older	98 [93, 105]	89 [82, 95]	1.95 [1.65, 1.95]
	*0*.*04* [*0*.*14*, −*0*.*10*]	*0*.*22* [*0*.*36*, *0*.*10*]	

*Note*: The corresponding logMAR scores are presented below in italics. Square brackets indicate the minimum and maximum scores across participants in each age group. CS scores for young and older participants correspond to median log units across all participants in each age group.

Abbreviations: CS, contrast sensitivity; HC, high contrast; LC, low contrast; VASs, visual acuity scores.

The study was approved by the local ethics committee at the College of Science and Engineering, University of Glasgow (approval no. FIMS00740), and conducted in line with the British Psychological Society ethics guidelines. Informed written consent was obtained from each participant before the study. Participants were compensated £6/h.

### 4.2 Stimuli

We used a set of 10 grayscaled, front‐view photographs of faces, oval cropped to remove external features, and pasted onto a uniform gray background (Gold, Bennett, & Sekuler, 1999). The pictures spanned 9.3 × 9.3° of visual angle; the face oval was 4.9 × 7.0° of visual angle. A unique image was presented on each trial by introducing phase noise (70% phase coherence) into the face images (Rousselet, Pernet, Bennett, & Sekuler, 2008). Noisy textures were created by fully randomizing the phase of the face images (0% phase coherence). All stimuli had the same amplitude spectrum, set to the mean amplitude of the face images. Face and noise images were revealed through “bubble masks,” that is, masks containing 10 two‐dimensional Gaussian apertures (σ = 0.36°), with the constraint that the center of the aperture remained in the face oval (Rousselet et al., 2014). We wrote our experiments in MATLAB using the Psychophysics Toolbox extensions (Brainard, 1997; Kleiner, Brainard, & Pelli, 2007; Pelli, 1997).

### 4.3 Procedure

Participants came in for two experimental sessions on separate days. During each session, we asked participants to minimize movement and blinking, or blink only when hitting a response button. We maintained a viewing distance of 80 cm using a chinrest.

In each experimental session, participants completed 12 blocks of 100 trials each, seated in a sound‐attenuated booth. The first block was a practice block of images without bubble masks. As such, across the two sessions participants performed 200 trials without bubble masks, and 2,200 trials with bubble masks. Practice blocks used a set of 10 face identities and 10 unique noise textures, each repeated five times were randomized within each block. Each practice session lasted about 60–75 min, including breaks, but excluding EEG electrode application.

On each trial, we instructed participants to categorize stimuli as fast and as accurately as possible, by pressing “1” for face, and “2” for texture on the numerical pad of a keyboard, using the index and middle finger of their dominant hand. Each trial began with a small black fixation cross (12 × 12 pixels, 0.4 × 0.4° of visual angle) displayed at the center of the monitor screen for a random time interval of 500–1,000 ms, followed by an image of a face or a texture presented for seven frames (~82 ms). After the stimulus, a blank gray screen was displayed until the participant responded. The fixation cross, the stimulus, and the blank response screen were all displayed on a uniform gray background with mean luminance of ~43 cd/m^2^. After each block of 100 trials, participants could take a break, and they received feedback on their performance in the previous block and on their overall performance in the experiment (median RT and percentage of correct responses). The next block started after participants pressed a key.

### 4.4 EEG recording and preprocessing

We recorded EEG data at 512 Hz using a 128‐channel BioSemi ActiveTwo EEG system (BioSemi, Amsterdam, the Netherlands). Four additional UltraFlat Active BioSemi electrodes were placed below and at the outer canthi of both eyes. Electrode offsets were kept between ±20 μV.

EEG data were preprocessed using MATLAB 2013b and the open‐source EEGLAB toolbox (Delorme et al., 2011; Delorme & Makeig, 2004). Data were first average referenced and detrended. Two types of filtering were then performed. First, data were band‐pass filtered between 1 and 30 Hz using a noncausal fourth‐order Butterworth filter. Independently, another dataset was created in which data were preprocessed with fourth‐order Butterworth filters: a high‐pass causal filter at 2 Hz and a low‐pass noncausal filter at 30 Hz, to preserve accurate timing of onsets (Acunzo, MacKenzie, & van Rossum, 2012; Luck, 2005; Rousselet, 2012; Widmann & Schröger, 2012).

Data from both datasets were then downsampled to 500 Hz, and epoched between −300 and 1,000 ms around stimulus onset. Mean baseline was removed from the causal‐filtered data, and channel mean was removed from each channel in the noncausal‐filtered data in order to increase the reliability of independent component analysis (ICA) (Groppe, Makeig, & Kutas, 2009). Noisy electrodes and trials were then detected by visual inspection of the noncausal dataset, and rejected on a participant‐by‐participant basis. Following visual inspection, one young participant and one older participant were excluded from further analyses due to noisy EEG signal. MI analysis confirmed the lack of sensitivity to any facial features in these participants. The resulting sample size was 17 young and 18 older participants. In this sample, more noisy channels were on average removed from older than from young participants' datasets (older participants: median = 10, min = 0, max = 24; young participants: median = 5, min = 0, max = 28; median difference = 4 [2, 7]). More noisy Bubble trials were also removed from older than from young participants' datasets (trials included in analyses, older participants: median 2,130, min 1987, max 2,180; young participants: median 2,178, min 2,023, max 2,198; median difference = 42 [23, 64]).

Subsequently, we performed ICA on the noncausal filtered dataset using the Infomax algorithm as implemented in the *runica* function in EEGLAB (Delorme & Makeig, 2004; Delorme, Sejnowski, & Makeig, 2007). The ICA weights were then applied to the causal filtered dataset to ensure removal of the same components, and artifactual components were rejected from both datasets (median = 4, min = 1, max = 27 for one older participant who displayed excessive blink activity; the second max was 17). Then, baseline correction was performed again, and data epochs were removed based on an absolute threshold value larger than 100 μV and the presence of a linear trend with an absolute slope larger than 75 μV per epoch and *R* ^2^ larger than .3. The median number of bubble trials accepted for analysis was, out of 1,100, for older participants: face trials = 1,069 [min = 999, max = 1,092]; noise trials = 1,067 [min = 986, max = 1,088]; for young participants: face trials = 1,090 [min = 1,006, max = 1,100]; noise trials = 1,089 [min = 1,014, max = 1,098]. Finally, we computed single‐trial spherical spline current source density waveforms using the CSD toolbox (Kayser, 2009; Tenke & Kayser, 2012). CSD waveforms were computed using parameters 50 iterations, *m* = 4, and *λ* = 10^−5^. The head radius was arbitrarily set to 10 cm, so that the ERP units are μV/cm^2^. The CSD transformation is a spatial high‐pass filtering of the data, which sharpens ERP topographies and reduces the influence of volume‐conducted activity. CSD waveforms are also reference free.

### 4.5 Electrode selection

We performed detailed analyses on the subset of four posterior midline electrodes that are sensitive to face features or conjunction of features: from top to bottom CPz, Pz, POz, and Oz (Rousselet et al., 2014; Schyns, Thut, & Gross, 2011). We only report the results of Oz because the other three electrodes had weak MI to face features in the two groups. We also selected two posterior‐lateral electrodes, one in the right hemisphere (RE), and one in the left hemisphere (LE) by measuring the difference between all bubble face trials and all bubble noise trials at all posterior‐lateral electrodes, squaring it, and selecting the left and the REs that showed the maximum difference in the period 130–250 ms. Across participants, the selected LE and RE were P7/8, or PO7/8, or their immediate neighbors. These electrodes are typically associated with large face ERPs in the literature.

### 4.6 Event‐related potentials

We compared the amplitude and latency of the N170 between the two age groups. To this end, we computed mean ERPs across trials for each participant, separately for face and noise trials, and for practice (without Bubbles) and regular (with Bubbles) trials. For ERPs recorded at the lateral‐occipital electrode in the right hemisphere (RE), we defined the N170 peak in individual participants as the minimum mean ERP between 110 and 230 ms, and considered separately its latency and amplitude.

### 4.7 Statistical analyses

We conducted statistical analyses using MATLAB 2013b and the LIMO EEG toolbox (Pernet et al., 2011). Throughout the paper, square brackets indicate 95% confidence intervals computed using the percentile bootstrap technique, with 1,000 bootstrap samples. Unless otherwise stated, median values are Harrell–Davis estimates of the second quartile (Harrell & Davis, 1982).

### 4.8 Mutual information

We used MI to quantify the dependence between stimulus features and behavioral and brain responses (Ince et al., 2017; Ince, Petersen, Swan, & Panzeri, 2009; Kayser, Ince, Gross, & Kayser, 2015; Park, Ince, Schyns, Thut, & Gross, 2015; Schyns et al., 2011). We binned pixel visibility across trials (due to random bubbles sampling), and behavioral and EEG responses into three equiprobable bins (for details, see Rousselet et al., 2014). We then calculated several MI quantities in single participants: MI(PIX; RT) to establish the relationship between image pixels and RTs; MI(PIX; CORRECT) to establish the relationship between image pixels and correct responses; MI(PIX; RESP) between pixels and response category; and MI(PIX; ERP) to establish the relationship between image pixels and ERPs. We computed these quantities separately for face and noise trials. To control for the variable number of trials in each participant arising as a result of EEG preprocessing, we scaled every MI quantity for every participant by a factor of *2Nln2* (Ince, Mazzoni, Bartels, Logothetis, & Panzeri, 2012), using the formula:

$${\mathrm{MI}}_{\text{scaled}}=\mathrm{MI}\times 2\times \mathrm{Nt}\times \log_{2},$$

where MI refers to mutual information values and Nt is the number of trials. MI_scaled_, therefore, reflects a measure of MI adjusted for a systematic upward bias in the information estimate that might arise due to limited data sampling, especially if the numbers of trials in the two age groups are systematically different. It also converts MI to be the effect size for a log‐likelihood test of independence (Sokal & Rohlf, 2012). All group‐difference analyses were performed using the scaled MI values.

### 4.9 MI: Classification images

We refer to MI between pixels and behavior or ERPs as classification images: they reveal the image pixels associated with modulations of the responses. We computed the MI(PIX, ERP) classification images at every time point within the first 400 ms following stimulus onset, using the noncausal and causal‐filtered datasets, and at each of the six electrodes specified above. We summarized each classification image with its maximum MI and reported these time courses per electrode.

#### 4.9.1 Single‐subject analyses

To establish the statistical significance of the classification image pixels while controlling for multiple comparisons arising from testing at multiple pixels, we performed a permutation test coupled with the threshold‐free cluster enhancement technique (Smith & Nichols, 2009) on individual participants' data (Rousselet et al., 2014).

### 4.10 Feature redundancy

We computed coinformation (coI) (Bell, 2003) (equivalent to interaction information (McGill, 1954) but with opposite sign for three variables) to quantify the triple dependence between eye visibility, RTs, and brain responses (Ince, 2017; Ince et al., 2017, 2016, 2015; Zhan, Ince, Van Rijsbergen, & Schyns, 2018), that is, coI(eye; RT; EEG). Positive values of coI quantify redundancy, specifically here redundant or overlapping information about the stimulus eye visibility, which is common to both EEG and RT responses. We computed coinformation redundancy with the following expression:

FeatRed=MI(EEG; eye visibility)+MI(RT; eye visibility−MI([EEG RT]; eye visibility)

We used here Gaussian‐Copula Mutual Information a semiparametric rank‐based estimator suitable for continuous variables (Ince et al., 2017) (see also Supplementary Methods) and considered a two‐dimensional EEG signal consisting of voltage together with its instantaneous temporal derivative (see Supplementary Methods).

If there is less information about eye visibility available from EEG and RT considered jointly, MI([EEG RT]; eye visibility), than there is when the two responses are considered independently, MI(EEG; eye visibility) + MI(RT; eye visibility), then this shows that part of the relationship quantified by MI(EEG; eye visibility) overlaps with that quantified by MI(RT; eye visibility). As such, redundancy quantifies the overlapping information content (i.e., a common driving effect) within both EEG and RT, about eye visibility. We computed this measure at all time points and electrodes for each participant independently. Then, for each participant, we plotted the time course of redundancy at occipital‐temporal electrodes specified above (i.e., RE and LE) between 0 and 400 ms poststimulus.

### 4.11 Group redundancy

We computed group‐level redundancy to quantify the degree to which median RT and individual peak redundancy time provide a common prediction of the age group (young vs. older) across individual participants. For these analyses, we quantized RT and peak redundancy time into three equally populated bins (splitting on tertiles of the distribution across participants) and calculated MI from the standard discrete definition (Cover & Thomas, 2005; Ince et al., 2017). We applied Miller–Madow bias correction to the individual MI estimated before calculating group redundancy as:

GroupRed=MI(FRP; group)+MI(RT; group)−MI([FRP RT]; group)

where FRP is feature redundancy peak time, RT is reaction time, and group is age group for each participant. We determined bootstrap confidence intervals via resampling participants with replacement 1,000 times.

### 4.12 ERP onset analyses

We quantified ERP onsets using the causal‐filtered datasets. To control for multiple comparisons, we used a bootstrap temporal clustering technique as implemented in LIMO EEG (Pernet et al., 2011; Pernet, Latinus, Nichols, & Rousselet, 2015).

#### 4.12.1 ERP_STD_ onset

To test whether age‐related delays reflect differences in the onset of afferent activity to the visual cortex, we looked at the time course of the *SD* across electrodes of the mean ERP (ERP_STD_). ERP_STD_ provides a compact description of the global ERP response, summarizing each participant's evoked brain activity across electrodes in one vector. This analysis was based on the notion that early visual activity can be characterized by a sudden increase in *SD* of the mean ERP across electrodes (Foxe & Simpson, 2002). We computed the ERP_STD_ time course for each individual participant and mean baseline centered it. Then, we localized the first peak the minimum height of which was five times the height of any peak in the baseline. Then, using ARESLab toolbox (Jekabsons, 2015), we built a piecewise‐linear regression model with three basis functions using the multivariate adaptive regression splines (Friedman, 1991) method. This approach divided the data into two segments and fitted each segment with separate models (regression splines). Onsets were defined as the location in time of the first knot of the fitted spline, that is, the point where division between the two models occurred.

#### 4.12.2 MI onset

We quantified MI onsets using the same technique as with ERP_STD_ onsets.

### 4.13 Topographic analyses

We computed topographic maps for each participant from the whole‐scalp MI(PIX; ERP) results, and for the whole‐scalp ERP results, at the individual MI peak, or ERP peak latency, respectively. Individual ERP topographic maps were squared. All topographic maps were interpolated and rendered in a 67 × 67 pixel image using the EEGLAB function *topoplot*, and then averaged across participants in each age group. Using the interpolated head maps, we then computed a hemispheric lateralization index for each participant using MI results. First, we normalized MI values in each participant between 0 and 1. Then, we saved the maximum pixel intensity in the left and the right hemisphere (lower left and right quadrants of the interpolated image), excluding the midline. Finally, we computed the lateralization index in each group as the ratio (MI_left_ − MI_right_)/(MI_left_ + MI_right_). Whole‐scalp MI was strongest at posterior‐lateral electrodes, and tended to be right lateralized in both groups (lateralization index for face trials, young = −0.18 [−0.31, −0.05]; older = −0.23 [−0.37, −0.09]; group difference = 0.07 [−0.07, 0.21]).

## Supporting information


**Appendix S1**: Supplementary Material

## Data Availability

A reproducibility package with data and code is available on figshare: https://doi.org/10.6084/m9.figshare.10744937. Single‐trial data from the young participants are available on Dryad: https://datadryad.org/stash/dataset/doi:10.5061/dryad.8m2g3
